# Augmentation effect of acupuncture on Bi’nao for hypophasis in patients with Bell’s palsy: study protocol for a randomized controlled trial

**DOI:** 10.1186/s13063-018-2699-z

**Published:** 2018-06-11

**Authors:** Xiaoyan Li, Chunlan Chen, Chuang Zhao, Zunyuan Li, Wei Liang, Zhidan Liu

**Affiliations:** 1 Department of Acupuncture, Baoshan Hospital of Integrated Traditional Chinese Medicine and Western Medicine, Shanghai, 201999 Shanghai China; 2 Department of Acupuncture, Shuguang Hospital Baoshan Branch, Shanghai University of Traditional Chinese Medicine, Shanghai, 201203 China

**Keywords:** Acupuncture, Hypophasis, Bell’s palsy, Facial paralysis

## Abstract

**Background:**

Hypophasis is one of the most frequently observed sequelae of patients with Bell’s palsy, who have not recovered completely, creating a clinical difficulty for physicians. Acupuncture therapy has been widely used to treat Bell’s palsy as a reasonable resolution for management of symptoms such as hypophasis. The number of acupuncture points (acu-points) is frequently selected in the approach of acupuncture therapy; however, whether these had high efficiency has not been proved. According to the literature review, Bi’nao was useful for treating eye and eye lipid diseases, which could be proved only by some successful cases. Thus, a randomized controlled trial was designed to evaluate the efficiency of the acu-point Bi’nao.

**Methods/Design:**

Participants with hypophasis as the major symptom are selected among patients with Bell’s palsy and randomly allocated into one of the three groups at a 1:1:1 allocation ratio. All participants receive conventional acupuncture therapy; however, those assigned to the real acupuncture group will be given added acupuncture therapy on the acu-point Bi’nao, while those assigned to the sham acupuncture group were given extra acupuncture therapy on the sham Bi’nao as a placebo. The efficacy of the acupuncture therapy on the acu-point Bi’nao for hypophasis will be evaluated by Eye Crack Width Measurement (ECWM) and Eyelid Strength Assessment (ESA) before and after therapy.

**Discussion:**

This is the first study assessing the safety and efficiency of Bi’nao in treating the hypophasis of patients with Bell’s palsy that might support the application of this acupuncture therapy. However, evaluating hypophasis is challenging, and, thus, ECWM and ESA were applied to measure the eyelid movement.

**Trial registration:**

Chinese Clinical Trials Registry, ChiCTR-INR-17012955. Registered on 12 October 2017.

**Electronic supplementary material:**

The online version of this article (10.1186/s13063-018-2699-z) contains supplementary material, which is available to authorized users.

## Background

Bell’s palsy is a type of self-limited peripheral neuropathy with uncertain pathogens. Patients with Bell’s palsy suffer from dysfunction of the facial nerve and inflexible muscle movements; the majority of them will recover within 1–3 months. However, a few of them will undergo a long recovery procedure and/or leave some sequelae such as deviation of mouth angle, hemifacial spasm, or hypophasis [1, 2].

Hypophasis is frequently observed among some patients with Bell’s palsy, who are almost at complete recovery, and is occasionally accompanied with a tiny droopy corner of the mouth, which is the major obstacle before full recovery from Bell’s palsy, as well as posing huge difficulties for doctors or rehabilitation physicians. The syndrome of hypophasis will continue to maintain the status quo unless appropriate therapy is applied; however, over-treatment such as excessive exercise or transcutaneous electrical nerve stimulation may lead to facial spasm [3, 4].

Acupuncture therapy has been widely used for the treatment of Bell’s palsy in China and worldwide [5–8]. Although the efficacy of the therapy has not been sufficiently proved by systematic reviews [9], its application has been considered a reasonable resolution for the management of some symptoms such as hypophasis. Although acu-points around the eye socket are frequently selected in the approach of acupuncture therapy, high-efficiency widely accepted acu-points have not yet been reported.

However, Bi’nao, an acupuncture point located on the large intestine meridian of the hand Yangming was found to be useful for treating hypophasis. The therapeutic effect of this acu-point was first reported in the book *Zhenjiu Jiayi Jing*, A-B classic of Acupuncture and Moxibustion [10]. Bi’nao is localized at the connection of the large intestine meridian of the hand Yangming and the collaterals of the hand Yangming. According to the records of *Huangdi Ming Tang Jing*, the collaterals of the hand Yangming is a critical joint of the small intestine meridian of the hand Taiyang, the bladder meridian of the foot Taiyang and Yangwei Channel. These four meridians are linked to the eye, and hence, acupuncture could be performed on Bi’nao for treating the eye and eye lipid diseases as described previously [11–19]. This acu-point was also used in our clinical practice for treating ophthalmoplegia and hypophasis; however, its efficacy could not be proved by a few successful cases. Thus, a randomized controlled trial (RCT) was designed to evaluate its efficiency compared to a placebo point.

## Methods/design

### Objective

The present study assessed the augmenting effect of acupuncture therapy on the acu-point Bi’nao for hypophasis through eye crack width and eyelid strength compared to a sham acu-point and no acupuncture treatment on Bi’nao.

### Design and setting

**Fig. 1:**
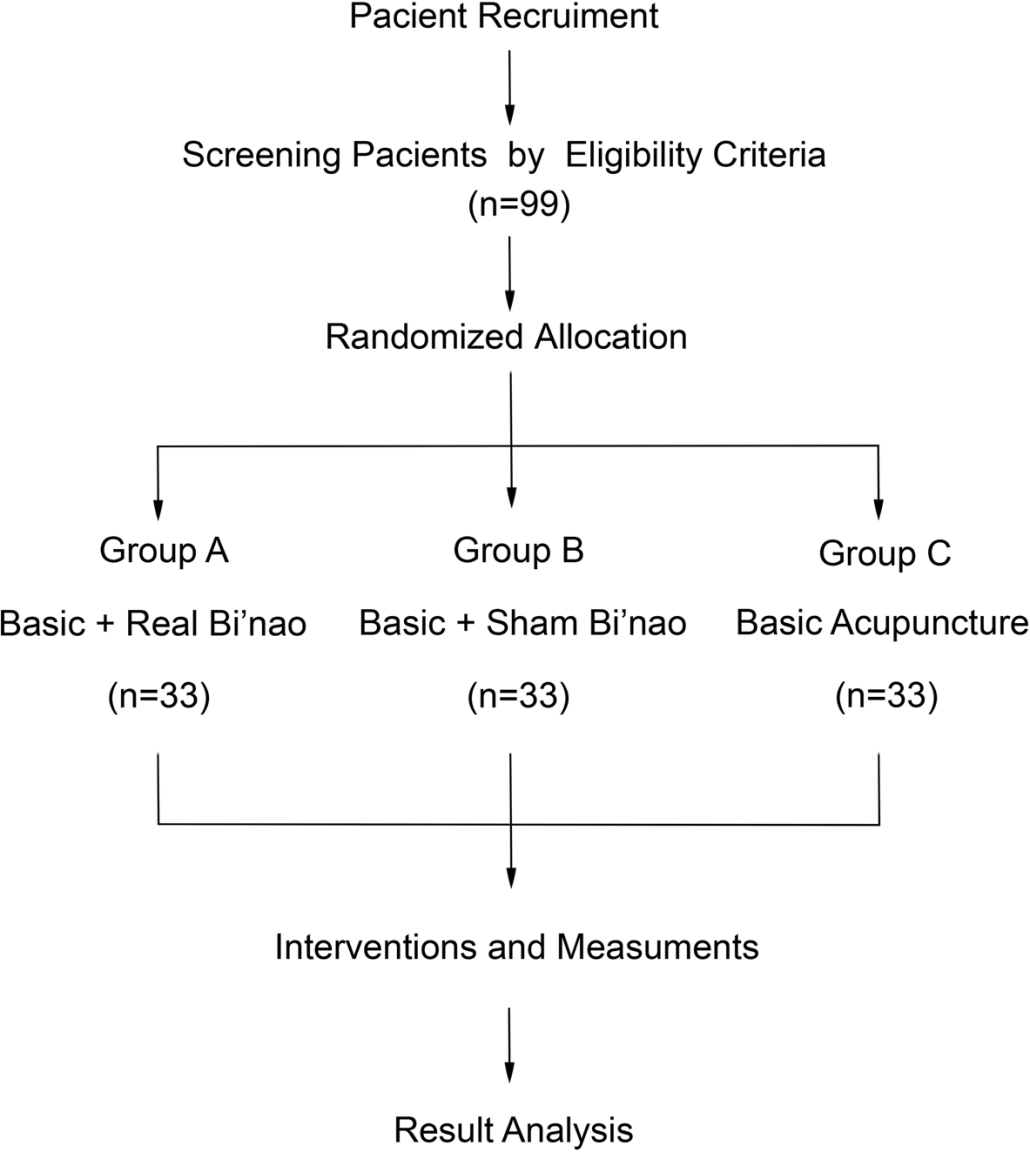
*Schematic* representation of the study design

This study is a single-center, three-arm, patient-blinded and assessor-blinded (to the type of acupuncture treatment), parallel-group, RCT conducted in China. The results followed the Consolidated Standards of Reporting Trials (CONSORT) guidelines as well as Standards for Reporting Interventions in Clinical Trials for Acupuncture (STRICTA) [20] and Standard Protocol Items: Recommendations for Interventional Trials (SPIRIT) guidelines [21]. This study (protocol version 1.0, 30 June 2017) has been registered at the Chinese Clinical Trials Registry (ChiCTR-INR-17012955). Figure 1 shows the research design and Additional file 1 displays the complete SPIRIT checklist.

### Recruitment period

The participants will be recruited from the Department of Acupuncture, Baoshan Hospital of Integrated Traditional Chinese Medicine and Western Medicine, Shanghai, China. The recruitment is expected to span from September 2017 to March 2020.

### Methods of recruitment

A total of 99 participants diagnosed with Bell’s palsy and mainly suffering the symptom of hypophasis through the outpatient or inpatient department will be recruited at the Department of Acupuncture, Neurology, and Geratology, Baoshan Hospital of Integrated Traditional Chinese Medicine and Western Medicine, Shanghai. The participants will be recruited by posters displayed in the hospital registration hall and brochures sent out to the visitors of those departments. The study title, research target, period, location, and contents of the intervention will be introduced to the participant.

### Study setting

The research assistants will obtain written consent (version 1.0, 30 June 2017) from the patients potentially eligible for the study. A researcher will explain the manipulation of acupuncture treatments used in traditional Chinese medicine (TCM) clinics. Subsequently, a researcher will screen whether the patient can participate in the study. If the patient fulfills the inclusion/exclusion criteria, a clinical research coordinator (CRC) specified by the scientific research department, Baoshan Hospital of Integrated Traditional Chinese Medicine and Western Medicine, Shanghai will contact an independent researcher who has the random number table; the participants will be randomly allocated into one of the three groups (group A, B, or C) at a 1:1:1 allocation ratio. All participants will receive conventional acupuncture therapy; however, the participants assigned to group A will be given additional acupuncture on the acu-point Bi’nao, while the participants assigned to group B will be given extra acupuncture on a point 1 cm backward to Bi’nao as a placebo point. Only group C participants will not receive extra acupuncture on any other acu-point.

The participants of this trial will undergo acupuncture treatments three times a week for one month. The Eye Crack Width Measurement (ECWM) and Eyelid Strength Assessment (ESA) of all the participants will be performed at the allocation time point, weekly during the treatment duration, and one month after the first therapy. The first ECWM and ESA value will be recorded as a baseline and the final values will be recorded as the end-time measurement. The follow-up visits for all participants will be scheduled at three months after the first acupuncture therapy. After the participants have completely recovered from hypophasis, the acupuncture therapy will be stopped and the final ECWM and ESA values will be recorded as an end-time measurement.

### Participants

The selected outpatients and inpatients are from the Departments of Acupuncture, Neurology, and Geratology of Baoshan Integrated Traditional Chinese Medicine and Western Medicine Hospital, Shanghai, China. Both male and female patients (age range = 18–60 years) are enrolled. All the patients are randomized into either Group A (acupuncture on the acu-point Bi’nao), Group B (placebo acu-point), or Group C (none acupuncture on the acu-point Bi’nao).

### Inclusion criteria

Patients with Bell’s palsy, who fulfilled the following inclusion criteria, will be enrolled in the study: unilateral facial paralysis; aged 18–60 years; Bell’s palsy onset > 1 month but < 3 months; most symptoms recovered but hypophasis was present; the distance between the upper and lower eyelids was > 2 mm; and those with negative pathological signs.

### Exclusion criteria

Participants meeting any of the following criteria will be excluded from the study: pregnant or breastfeeding; woman of child-bearing age during the study period; patients who had received medicine or acupuncture therapy in other hospitals before this study; diabetes course ≥ 5 years, grade 3 hypertension or hyperlipidemia ≥ 3 years; history of malignant tumors, sexually transmitted disease, chronic renal or hepatic disease, glaucoma, acute otitis, ipsilateral chronic otitis, tuberculosis, immunodeficiency syndromes, recent head injury, psychiatric disease, infectious diseases, or any other conditions that present risk of being influenced by the study treatment or that may affect the completion of the study; and patients participated in other clinical trials within three months.

### Randomization and blinding

**Fig. 2:**
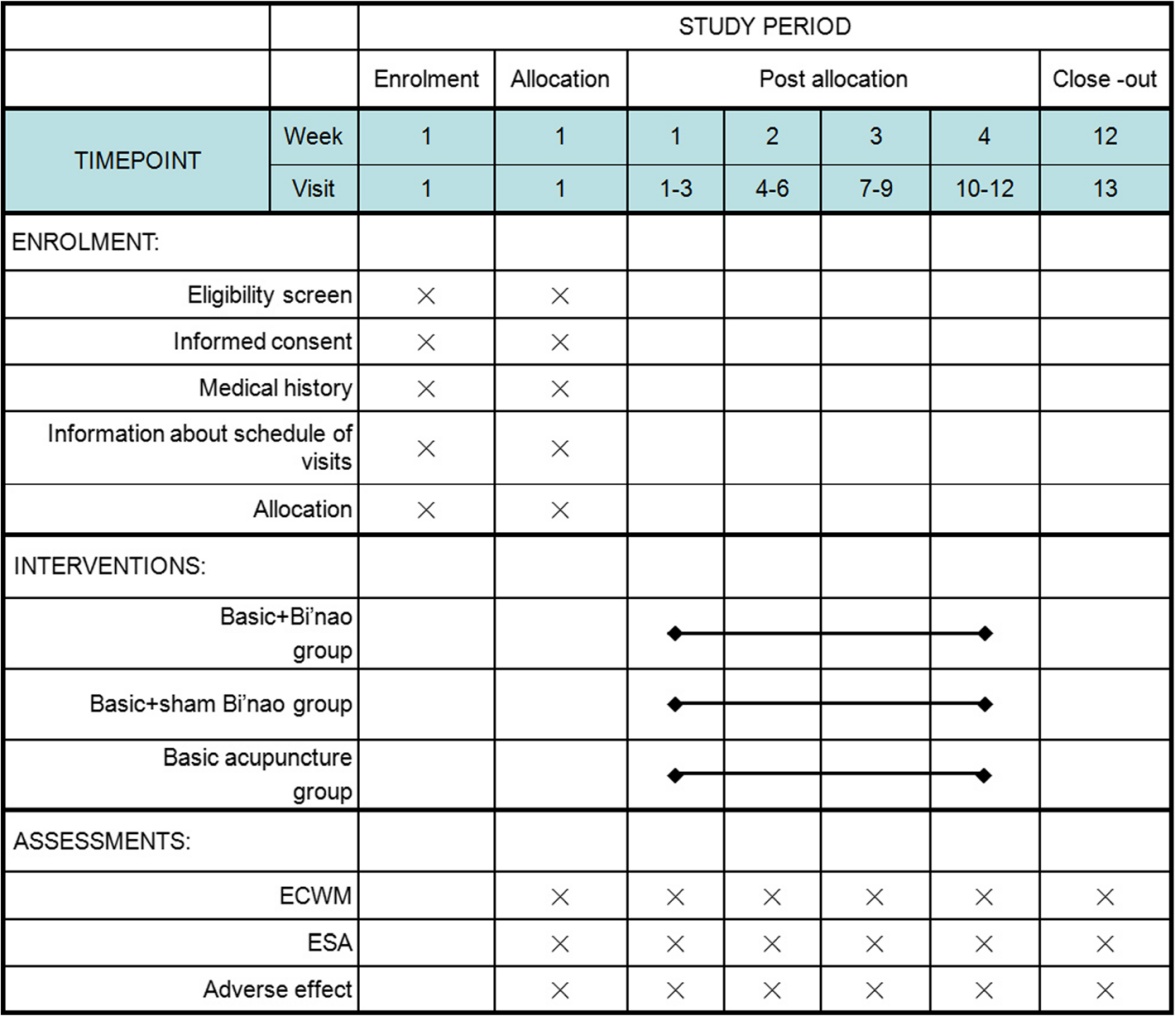
The Standard Protocol Items: Recommendations for Interventional Trials (SPIRIT) Figure. The schedule of enrollment, allocation, interventions, and assessments. ECWM Eye Crack Width Measurement, ESA Eyelid Strength Assessment

Figure 2 illustrates the schedule of enrolment, allocation, interventions, and assessments in this trial. The enrolled participants will be randomly assigned to group A, B, or C (1:1:1). An independent statistician will generate the block randomization scheme in a blinded manner. The table will be managed by another independent researcher who is not involved in the recruitment, acupuncture treatment, or assessment. The CRC will send the assignment information to the researcher who will conduct the random allocation; subsequently, the researcher will only provide assignment information to the doctors of TCM, who will perform the acupuncture treatment. In order to ensure concealing of the allocation, the information will be recorded in an allocation log by the researcher and not opened until the data are locked. The participants will be blinded to the type of acupuncture treatment; the assessor, data managers, statisticians, and study monitors will be blinded to the allocation. The blinding will be maintained until the data are locked. For evaluation in a blinded manner, the allocation guessing will be assessed immediately after the final treatment. The practitioners and assessors will be instructed to treat the participants according to the predefined standard operating procedures (SOPs) during the trial to maintain blinding; un-blinding will be considered only in the case of rights and safety of the participants, including the occurrence of serious adverse effects (SAEs). In such cases, the researchers will notify the principal investigator (PI) and document the subsequent processes.

### Intervention

#### Acupuncture treatment

Acupuncture will be performed by specialists in TCM and follow the STRICTA 2010 checklist (Table 1).

**Table 1:** Details of acupuncture intervention (according to the Standards for Reporting Interventions in Clinical Trials of Acupuncture (STRICTA) 2010 guidelines Expansion of Item 5 from Consolidated Standards of Reporting Trials 2010 guidelines)

Acupuncture rationale	Style of acupuncture	Traditional Chinese Medicine
	Rationale for treatment	Acupuncture has been historically used to treat hypophasis in facial palsy. Additionally, it is known to be a safe treatment used in a wide range of symptoms caused by Bell’s palsy
	Extent to which treatment varied	The participants in the intervention group all receive the same manual acupuncture treatment
Details of needling	Number of needle insertions per individual per session	8–9
	Names of the insertion points (uni/bilateral)	BL2, GB14, SJ23, Shangming, LI14 (unilateral, affected side); SJ5, KI6(bilateral)
	Depth of insertion	10–30 mm (exact depth shown in Table 2)
	Response sought	De-qi
	Needle stimulation	Manual stimulation (exact details are in the text)
	Needle retention time	20 min
	Needle type	0.30 mm (diameter) × 25 mm (length) disposal needle (Huatuo Acupuncture Inc., Suzhou, China)
Treatment regimen	Number of treatment sessions	12
	Frequency and duration of treatment sessions	3 sessions per week for 4 weeks
Other components of treatment	Details of other interventions administered to the acupuncture group	No other interventions are done
	Setting and context of treatment	All participants are informed that they will receive acupuncture treatment, which can potentially reduce symptoms of hypophasis; however, the control group will have to complete the evaluations during the first week before receiving the same treatment as the acupuncture group
Practitioner background	Description of participating acupuncturists	Specialists in TCM with at least 3 years of practice in acupuncture
Control or comparator interventions	Rationale for the control or comparator in the context of the research question	Sham acu-point is used as a placebo control
	Precise description of the control or comparator	Placebo control group be given extra acupuncture on a point 1 cm backward to Bi’nao as a placebo point

**Fig. 3:**
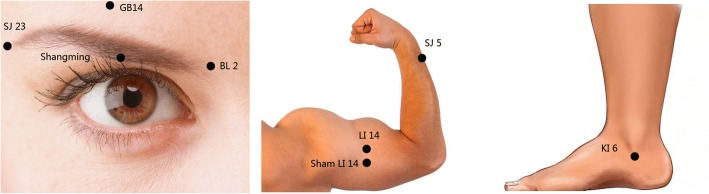
Location of acupoints. BL2, Cuanzhu, on the medial end of the eyebrow; GB14, Yangbai, directly above the pupil, 1 cun above the midpoint of the eyebrow; Shangming, in the orbital cavity and at the midpoint of the orbital margin; SJ23, Sizhukong, in the depression at the lateral end of the eyebrow; LI14, Bi’nao, at the stop of deltoid muscle on the line between LI15 (Jianyu) and Quchi (LI11), 7 cun above Quchi (LI11); Sham LI14, 1 cm backward to Bi’nao; SJ5, Waiguan, on the line joining Yangchi (SJ4) and the tip of elbow, 2 cun above the dorsocarpal transverse crease between the ulna and radius; KI6, Zhaohai, in the depression directly below the tip of the medial malleolus

All patients from the three groups will undergo acupuncture therapy at Cuanzhu (BL2), Yangbai (GB14), Sizhukong (SJ23), Shangming, Waiguan (SJ5), and Zhaohai (KI6) points (Fig. 3) as basic therapy. The acu-points are identified by the Method of Point Location issued by the World Health Organization (WHO). The acupuncture needles will be inserted straightly or obliquely at a depth of 10–30 mm to effectuate the desired sensation of “De qi” (Table 2) by reinforcing-reducing techniques of lifting, thrusting, twisting, and rotating the needles moderately. When the acupuncture doctor feels sinking, astringent, and tightness around the needle by his/her finger while the patient senses soreness, numbness, distension, and heaviness around the acu-point, this will be considered the arrival of the “De qi” sensation [22]. All the needles will be retained for 20 min. The acupuncture therapy will be performed three times each week on Monday, Wednesday, and Friday. Consequently, the participants in Group A (real acu-point group) will receive extra manual acupuncture therapy at Bi’nao (LI14) and participants in Group B (sham acu-point group) will receive extra manual acupuncture therapy three times a week as Group A but 1 cm backward to Bi’nao (Fig. 3) as a placebo point. Participants in Group C will receive no extra acupuncture therapy except basic therapy. The treatment will be performed for one month and all the participants will be reminded of the next visit by researchers.

**Table 2:** Acu-points and needle insertion procedure

Acupuncture point	Direction	Depth (mm)
BL2 (Cuanzhu, affected side)	Transversely, along with geisoma	5–10
GB14 (Yangbai, affected side)	Transversely, toward geisoma	5–10
SJ23 (Sizhukong, affected side)	Obliquely toward partes temporalis	5–10
Shangming (affected side)	Transversely, along with gap between the eye socket and the eyeball	5–10
LI14 (Bi’nao, affected side)	Perpendicular to the skin	20–30
SJ5 (Waiguan, bilateral)	Perpendicular to the skin	10–20
KI6 (Zhaohai, bilateral)	Perpendicular to the skin	5–10

#### Allowed or prohibited concomitant treatment

The use of medications or other therapies for hypophasis will be discouraged during this trial. The treatments of individuals for other underlying chronic diseases will be allowed if they do not change during the study period and will be recorded accordingly, including the name, dosage, and duration of the medication. If a violation occurs during the study period, the patient will be excluded from the study.

### Outcome measures

#### Primary outcome

ECWM will be performed using a graduated ruler to measure the width (mm) between the upper and lower eyelids when the patients attempt to close his eyes. The measurements will be taken three times and the average recorded. ECWM will be performed before acupuncture therapy as the primary time measurement, weekly during the treatment duration, and at the first month after the first therapy by three physicians, and the average value recorded. The mean value and standard deviation among the participants in every group will be calculated; the differential values of eye crack width (mm) between the intervention and control groups will be compared.

#### Secondary outcome

ESA [23] will be conducted based on the muscle strength when the eyes are closed. The evaluator places the index finger on the patient’s upper eyelids and the patient will be required to close his eye. The difference in the muscle strength between the two eyelids will be recorded according to the following standard: level 5, normal contraction in affected side, which is symmetrical with the healthy side; level 4, approximately normal contraction and slightly asymmetric with healthy side; level 3, strength is approximately half of the healthy side; level 2, strength is approximately one-quarter of the healthy side; level 1, a slight muscular contraction in the affected side; level 0, no sign of muscle contraction in the affected side. Assessment will be performed at the time points mentioned above and at the first month after the therapy by three physicians, and the average value will be recorded. Levels 0–5 will be endowed with numeric values 0–5 as the ESA score; the average score among the participants in each group will be calculated. The mean value and standard deviation of ESA scores between the intervention and control groups will be compared.

#### Follow-up

All participants will be followed up at three months after the first acupuncture therapy, including those who completely recovered from hypophasis within one month. The follow-up will be accomplished by a clinic visit or telephone survey about the progress and recurrence of hypophasis.

#### Quality control

All acupuncturists and assessors will be required to undergo specialized training before the trial in order to guarantee consistent practices. The training program will include diagnosis, inclusion and exclusion criteria, the location of the acu-points, acupuncture manipulation techniques, and completion of the case report forms. Dropouts and withdrawals from the study will be recorded based on the intervention and follow-up periods. This trial will be monitored by the Scientific Research Department of Baoshan Hospital of Integrated Traditional Chinese Medicine and Western Medicine, Shanghai. A qualified clinical research investigator will be specified by the department office and periodic monitoring will guarantee accuracy and quality throughout the study.

### Withdrawal and dropout

All participants will have the right to withdraw from this study at any time. Participation will be terminated at any stage if the individual refuses to continue, withdraws consent, or violates the inclusion or exclusion criteria. The investigator may also exclude the participants from the study in order to protect their safety and/or if they are unwilling or unable to comply with the required study procedures after consultation with the institutional review board (IRB).

### Safety issues

Adverse events (AEs) will be recorded using the standard operating procedures for monitoring and reporting all AEs, which refer to all the harmful and unintended reactions that occur during the acupuncture treatment. The symptoms, date of occurrence, disappearance, causality, and severity of the AEs will be recorded. The putative AEs of acupuncture include the following: local bleeding or hematoma; more than slight sharp pain; aggravation of symptoms; syncope; nausea; headache; peripheral nerve injury; and infection. Participants will be informed of the potential AEs in advance.

During the study period, all researchers will ensure patient safety. When SAEs occur, the researchers will immediately halt the study and promptly provide appropriate management. The PI will also report the AEs to the IRB and halt the study partially or completely until further notice. Then, the IRB will determine whether the clinical study can be continued. The trial will be stopped if the IRB speculates unacceptable risks of SAEs. The hospital provides insurance coverage for harm associated with the interventions during this trial as necessary.

### Statistical methods

#### Sample size calculation

The enumeration data of parallel-controlled trial (1:1:1) are analyzed statistically using two-sided superiority test, where α = 0.05, power = 90%. The formula used for the calculation was as follows:

$$ n\kern0.5em =\kern0.5em \frac{{\left({Z}_{1-a}+{Z}_{1-\upbeta}\right)}^2\kern0.5em \times \left({\sigma}_1^2+{\sigma}_2^2\right)}{\delta^2} $$

The change in the standard deviation of ECWM score before and after the treatment in the control group was presumed as 1 point and that in the treatment group as 3 points. This will be considered clinically significant if the change in the ECWM score before and after the treatment in the treatment group is 2 points higher than that in the control group, and the sample size was estimated as follows:

$$ n\kern0.5em =\kern0.5em \frac{{\left({Z}_{1-a}+{Z}_{1-\upbeta}\right)}^2\kern0.5em \times \left({\sigma}_1^2+{\sigma}_2^2\right)}{\delta^2}\kern0.5em =\kern0.5em \frac{{\left(1.96+1.28\right)}^2\kern0.5em \times \left({1}^2+{3}^2\right)}{2^2}\kern0.5em =\kern0.5em 26 $$

Thus, a minimum of 27 patients was estimated in each group. The dropout rate is considered to be 20%; therefore, at least 33 patients should be enrolled in each group. Hence, a total of 99 patients are required to conduct this study.

#### Statistical analysis

In this study, rather than the investigator, professionals will analyze the data using intention-to-treat and per-protocol approaches. The demographic and clinical characteristics of the participants (such as sex and age) will be processed based on descriptive analyses. The ECWM values and ESA scores will be compared as a baseline measurement. The quantitative data will be presented as average, standard deviation, median value, and range, while the qualitative data will be presented as the frequency and percentage.

For the primary and secondary outcome measurements, one-way ANOVA test will be applied to identify the differences between the of ECWM values and ESA scores among the groups. In case of abnormally distributed data, Kruskal–Wallis H test will be performed.

All AEs reported during the study will be included in the clinical report; then, the prevalence of AEs will be calculated. The percentage of participants with AEs in each group will be calculated and compared using the chi-squared or Fisher’s exact test. Statistical analyses will be performed using IBM SPSS Windows version 19.0 (IBM Co., Armonk, NY, USA). The tests were two-sided; a *P* value < 0.05 will be considered statistically significant.

### Data collection, management, and protection

Data will be collected by a specialized research assistant, who will be responsible for the patient consents and case report forms. Soft copies of the research materials will be saved on a storage device not connected to the Internet. All data will be maintained in a locked storage and only those with permission from the PI will be able to access the data. All the retained data will be coded to identify the patients instead of personal information.

The IRB will oversee the intra-study data sharing process, with input from the Data Management Subcommittee. The PIs will be given access to the cleaned datasets. In order to ensure confidentiality, the project team members will be blinded to any identifying participant information.

### Monitoring

To ensure that the quality of the data are in accordance with the predetermined protocol and SOPs, the regular day-to-day monitoring will be carried out by a qualified clinical research investigator specified by the Scientific Research Department of Baoshan Hospital of Integrated Traditional Chinese Medicine and Western Medicine, Shanghai. The investigator will be blinded to the allocation and will examine whether the recruitment procedures and data recording followed the protocol in the case report forms. The investigator will discuss the unpredictable changes in the research process, such as changes to the eligibility criteria, treatment regimens, or duration of follow-up with independent researchers and statisticians. In the case of SAEs or crucial issues, the investigator will determine whether the events are acceptable or whether it is necessary to change or terminate the trial.

Thus, the investigator will oversee the compliance of the study protocol and informed consent documents, supervise the trial progress, participant recruitment, data quality, timeliness, the performance of the interventions, and all fields and process of the trial. If any important protocol modifications or violations arise during the trial, they will be handled by an independent researcher, who is responsible for preparing an amended protocol and resubmission to the IRB for a final decision under the direction of the IRB.

### Ethics and dissemination

The study was planned in accordance with the Helsinki Declaration and the Korean Good Clinical Practice Guidelines to protect the participants and was approved by the Medical Ethics Committee Board of Baoshan Hospital of Integrated Traditional Chinese Medicine and Western Medicine, Shanghai (201709–02). The participants will be informed of the potential benefits, risks, alternatives, and responsibilities of the study by the researchers during the consent process. They will be fully aware that they are free to withdraw from the study at any time. Any AEs (described as unfavorable or unintended signs, symptoms, or diseases occurring after treatment) related to acupuncture treatment will be observed and reported by patients and practitioners during each patient visit. In addition, all vital signs and AEs will be measured and recorded during each visit. The findings will be disseminated in peer-reviewed journals and conference presentations.

## Discussion

The present study evaluated the efficacy of acupuncture therapy on the acupoint Bi’nao for hypophasis in patients with Bell’s palsy based on the previous studies and our clinical experience. When planning a manual acupuncture study, the duration of treatment, treatment points, and frequency of stimulation should be considered as these can affect the outcomes of acupuncture. However, in a RCT, only one variable could be tested. Thus, we established this regimented acupuncture protocol that has been discussed and optimized at the Baoshan Hospital of Integrated Traditional Chinese Medicine and Western Medicine, Shanghai.

In addition, the function of “De qi” will be considered in the trial; it is based on subjective reporting of the patient (soreness, numbness, fullness, radiating sensation) and is regarded as a sign of efficacy according to TCM. A majority of the contemporary acupuncturists still seek “De qi,” rendering it as fundamental to efficacy. The placebo effect produced by sham acupuncture was considered to influence the disease lightly, although needle pricking might induce non-specific physiological reactions. The sensation of “De qi” can be accurately described by sense felt by either the practitioner or patient, and reliable scales such as the Massachusetts General Hospital Acupuncture Sensation Scale (MASS) have already been applied in clinics measuring the intensity of “De qi” sensation [24–26].

The assessment of hypophasis is rather challenging due to the lack of a widely recognized method or scale. The House-Brackmann scale and Sunnybrook Grading System are suitable for the overall assessment of Bell’s palsy, but are not applicable to individual symptoms such as hypophasis. Thus, we created the ECWM and ESA to evaluate eyelid movement according to some neurological function classification. Although this was an innovative approach, it was also a limitation of this study.

The results showed that the acupuncture treatment on the acu-point Bi’nao is a safe and effective method for improving hypophasis. Furthermore, the study provided evidence to support the application of acupuncture therapy in the management of Bell’s palsy and its sequelae in the future.

### Trial status

This trial began in September 2017 after IRB approval. Trial completion is expected by the end of June 2020. Recruitment is currently ongoing.

## Additional file

Additional file 1: A complete SPIRIT checklist. (DOC 122 kb)

## Abbreviations

AE: Adverse event
CONSORT: Consolidated Standards of Reporting Trials
CRC: Clinical research coordinator
ECWM: Eye Crack Width Measurement
ESA: Eyelid Strength Assessment
IRB: Institutional review board
SOP: Standard operating procedure
SPIRIT: Standard Protocol Items Recommendations for Interventional Trials
STRICTA: Standard for Reporting Interventions in Clinical Trials for Acupuncture
TCM: Traditional Chinese medicine
